# Job vacancies at ECDC

**DOI:** 10.2807/1560-7917.ES.2022.27.38.220922v2

**Published:** 2022-09-22

**Authors:** Eurosurveillance editorial team

**Affiliations:** 1 European Centre for Disease Prevention and Control (ECDC), Stockholm, Sweden

The European Centre for Disease Prevention and Control (ECDC) invites applications for the following positions:

 

- **Knowledge Management Officer**
  The application deadline expires on 18 October 2022.

 

- **Data Scientist**
  The application deadline expires on 20 October 2022.

 

For more information, please visit the ECDC website: 

https://erecruitment.ecdc.europa.eu/?page=advertisement

